# Not all weeds are created equal: A database approach uncovers differences in the sexual system of native and introduced weeds

**DOI:** 10.1002/ece3.2820

**Published:** 2017-03-18

**Authors:** Megan L. Van Etten, Jeffrey K. Conner, Shu‐Mei Chang, Regina S. Baucom

**Affiliations:** ^1^ Ecology and Evolutionary Biology DepartmentUniversity of MichiganAnn ArborMIUSA; ^2^ Kellogg Biological StationMichigan State UniversityHickory CornersMIUSA; ^3^ Department of Plant BiologyUniversity of GeorgiaAthensGAUSA

**Keywords:** Baker's law, ecological filtering, invasive, sexual system, weed

## Abstract

Weedy species provide excellent opportunities to examine the process of successful colonization of novel environments. Despite the influence of the sexual system on a variety of processes from reproduction to genetic structure, how the sexual system of species influences weediness has received only limited consideration. We examined the hypothesis that weedy plants have an increased likelihood of being self‐compatible compared with nonweedy plants; this hypothesis is derived from Baker's law, which states that species that can reproduce uniparentally are more likely to successfully establish in a new habitat where mates are lacking. We combined a database of the weed (weedy/nonweedy) and introduction status (introduced/native) of plant species found in the USA with a database of plant sexual systems and determined whether native and introduced weeds varied in their sexual systems compared with native and introduced nonweeds. We found that introduced weeds are overrepresented by species with both male and female functions present within a single flower (hermaphrodites) whereas weeds native to the USA are overrepresented by species with male and female flowers present on a single plant (monoecious species). Overall, our results show that Baker's law is supported at the level of the sexual system, thus providing further evidence that uniparental reproduction is an important component of being either a native or introduced weed.

## 1 Introduction

Individuals colonizing a new habitat often face the fundamental problem of a lack of mates. Baker hypothesized that species with the ability to reproduce uniparentally are more likely to successfully colonize new areas compared with species that rely on mates for propagation (Baker, 1955). While the scenario of island colonization and establishment originally influenced his idea, he later applied this concept to the evolution of agrestals and ruderals, or plants that colonize agricultural fields or waste areas such as roadsides, respectively (Baker, 1965). He examined the Asteraceae family, which contains both highly selfing and self‐incompatible species and found that the weedy species in this group were typified by rapid development, rapid flowering, increased plasticity, and self‐compatibility (Baker, 1965). Baker's key insight that the ability to colonize was related to uniparental reproduction when mates are lacking—formally known as Baker's law—is now a cornerstone hypothesis that is supported by studies of plants and animals and has been examined in a variety of contexts, for example, island colonization, metapopulation dynamics, invasive species, and range expansion (reviewed in Pannell & Barrett, 1998; Pannell et al., 2015). Strikingly, although Baker's law has a broad reach in ecology and evolution, few explicit tests examine the hypothesis that weeds (broadly described as plants that are found in places they are not wanted; Radosevich, Holt, & Ghersa, 2007) exhibit an increased capacity for self‐fertilization and/or uniparental reproduction.

The available examinations of Baker's law as it applies to weeds consider the frequency of self‐compatibility in invasive plants, which are plants that have been introduced to new areas and are subsequently deemed problematic. These comparisons provide support for Baker's law, but are limited to particular taxonomic groups (Iridaceae: Van Kleunen, Manning, Pasqualetto, & Johnson, 2008; Asteraceae: Hao, Qiang, Chrobock, van Kleunen, & Liu, 2011), a restricted geographic region (South African invasives: Rambuda & Johnson, 2004; European invasives: Van Kleunen & Johnson, 2007), or work that compares invasives (hereafter “introduced weeds”) to native species (Burns, Ashman, Steets, Harmon‐Threatt, & Knight, 2011). While it is surprising that few studies of introduced weeds consider Baker's law—especially since much of its development centered around weedy plants—it is also remarkable that there are no large‐scale examinations, to our knowledge, of the potential that native weeds (plants that are native to a particular habitat and deemed weedy or problematic) are more likely to exhibit an increased capacity for self‐fertilization compared with native nonweeds. There are certainly similarities between introduced and native weeds that would suggest the ability to self would likewise be favored in native weeds—both types of weeds exhibit the typical set of “weediness traits,” for example, high fecundity, annual life form, and rapid growth in comparison with introduced and native nonweeds (Kuester, Conner, Culley, & Baucom, 2014).

The processes that lead to “weediness” in these two classes of weeds, however, are potentially very different. For example, introduced species are accidentally or purposefully moved across great distances, and those that establish in new areas may or may not eventually become weedy or invasive (Richardson, Pyšek et al., 2000; Williamson, 1996). Those that do become weedy are hypothesized to exhibit preexisting traits that allow for uniparental reproduction in mate‐limited areas (Pannell, 2015) and therefore may show a strong pattern of enrichment for hermaphroditism. In comparison, native weeds may not necessarily travel long distances (Valéry, Fritz, Lefeuvre, & Simberloff, 2008) and may or may not be expected to experience a lack of mates that is as extreme as that envisioned for introduced species and therefore may show enrichment for sexual systems that provide other benefits (such as a reduction in selfing rates). However, native weeds are generally known to be good colonizers of altered or disturbed habitats (Simberloff, Souza, Nuñez, Barrios‐Garcia, & Bunn, 2012), and for this reason may likewise exhibit reproductive traits that preadapt them for successful establishment in newly disturbed areas. Thus, different factors may be involved in the pathway to “weediness” for native and introduced species, which may or may not result in different sets of traits being important to their success.

Furthermore, while weediness is known to be associated with a broad set of traits (Kuester et al., 2014 and references therein), the potential that particular sexual systems are enriched in weedy plants compared with nonweeds has yet to be comprehensively examined in any flora. Plants that have both male and female organs within the same flower (hermaphrodites), or on separate flowers of the same plant (monoecy, andromonoecy, and gynomonoecy), are more likely to produce progeny in mate‐limited areas compared to species with separate sexes (dioecy, androdioecy, gynodioecy). We would thus predict, based on Baker's law, that weedy plants may be enriched for hermaphrodites or monoecious species compared with nonweeds as a mechanism of ensuring uniparental reproduction.

Here we test this prediction by combining two existing databases: a recently published Sex Systems Database (Tree of Sex Consortium, 2014) with a database of plant species found within the USA for which the weed (weedy vs. nonweedy) and introduction status (native vs. introduced) are known (database from Kuester et al., 2014). We used this concatenated database to test for an association between sexual system (hermaphrodite, monoecy, gynomonoecy, andromonoecy, dioecy, androdioecy) and weediness status for both native and introduced species. We used both taxonomic comparisons and comparisons that control for phylogenetic relatedness to test for enrichment. Our broad expectations are that both native and introduced weeds should have a larger proportion of hermaphrodites and/or monoecious species than nonweeds as these sexual systems increase the likelihood of uniparental reproduction.

## 2 Materials and Methods

The Sex Systems Database (Tree of Sex Consortium, 2014) was concatenated with a database of North American plant species that included introduction status (native/introduced) and weed status (weedy/nonweedy) (Kuester et al., 2014), in which weeds were defined as troublesome plants in agriculture, horticulture, ornamental, and natural areas. Sexual systems with very few occurrences were removed (androdioecy, apomictic, gynomonoecy, other, polygamomonoecy; *N* = 12). Many of the species in the Sex Systems Database were not in the Weed Database, leaving 1,077 species for further analyses. No statistical difference was found in the distribution of sexual systems between the full Sex Systems Database and the merged database (*χ* ^2^=0.09, *df* = 5, *p* = 1.0); thus, the merged dataset is representative of the Sex Systems Database.

To determine the effect of sexual systems (see Table 1) on weed status, we performed binomial regressions separately for each sexual system and weed status comparison. For example, using a dummy variable of hermaphroditic or not hermaphroditic as the predictor and introduced weed status (nonweedy vs. weedy) as the dependent variable in a binomial regression, we tested whether there was a difference in the probability of being a weed between hermaphroditic and nonhermaphroditic species. Separate regressions were run for native and introduced species. We also performed a multinomial regression including all the sexual systems in Table 1, but because results from multinomial logistic regression are difficult to interpret and were qualitatively the same as the binomial regressions, we chose to report results from the binomial tests (see Sup Table 1 for multinomial regressions). All analyses were performed in R (R Core Team, 2014).

**Table 1:** Description of sexual systems in this study, including written description, symbolic description (parentheses denote a single plant), and the ability to possibly self‐fertilize

	Description	Symbolic description	Possibly able to self with one plant?
Hermaphrodite (*N* = 662)	Male and female function within a single flower	(⚥)	Y
Dioecy (*N* = 182)	Male and female function on different plants	(♀) + (♂)	N
Monoecy (*N* = 145)	Male and female function in separate flowers on a single plant	(♀♂)	Y
Gynodioecy (*N* = 47)	Female plants and hermaphrodite plants	(♀) + (⚥)	Y, for ⚥
Polygamodioecy (*N* = 25)	Male and hermaphrodite‐flowered plants and female and hermaphrodite‐flowered plants	(⚥♂) + (♀⚥)	Y
Andromonoecy (*N* = 16)	Male and hermaphrodite flowers on a single plant	(⚥♂)	Y

Preliminary examination of the database indicated that sexual systems were relatively conserved within genera but varied among genera (between 1 and 6 sexual systems present per family within the database). Thus, we performed a phylogenetic logistic regression (Ives & Garland, 2010) to determine whether results from the above taxonomic comparison were influenced by shared evolutionary history. This method is similar to a normal logistic regression except a phylogenetic signal is added to the model (called alpha) to allow the detection of main variable effects while accounting for phylogeny. A value of α > −4 suggests a detectable phylogenetic signal. We created a genus level tree (i.e., all species within a genus were polytomies) using phylomatic V3 (Webb & Donoghue, 2004) and the R20120829 megatree (Available at: https://github.com/camwebb/tree-of-trees/blob/master/megatrees/R20120829.new), which resulted in a tree containing 1,071 species (335 introduced species and 735 native species; six species were not found on the megatree) in 194 genera. An ultrametric tree with time‐scaled branches was created using the wikstrom.ages file and the bladj procedure in phylcom‐4.2 (Webb, Ackerly, & Kembel, 2008). We used the phylolm package in R (Si, Ho, & Ane, 2014) to perform the phylogenetic logistic regression analysis for the three most common sexual systems (*i.e*., hermaphroditism, dioecy, and monoecy) for introduced and native species separately. We performed this analysis on these three common sexual systems as the others were represented by five species or less per weed/nonweed comparison, and preliminary results from the logistic regressions of all sexual systems indicated there were no differences between weeds and nonweeds for gynodioecy, polygamodioecy, and andromonoecy.

## 3 Results

**Figure 1:**
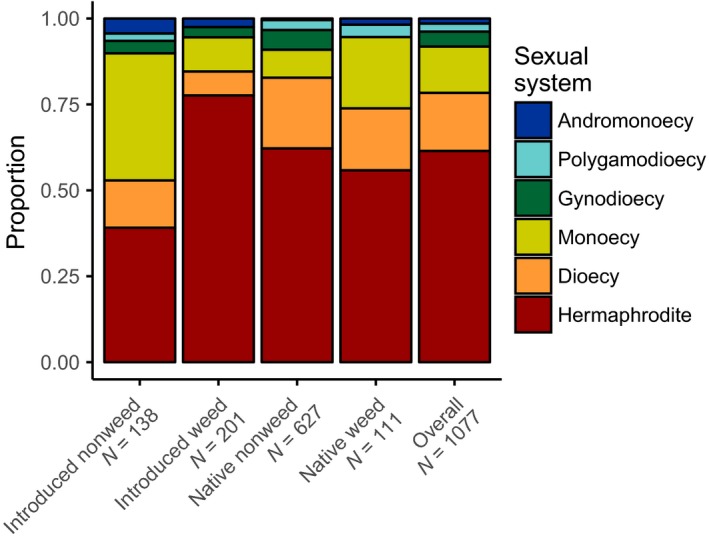
The proportion of each sexual system shown according to introduction and weed status combination

**Figure 2:**
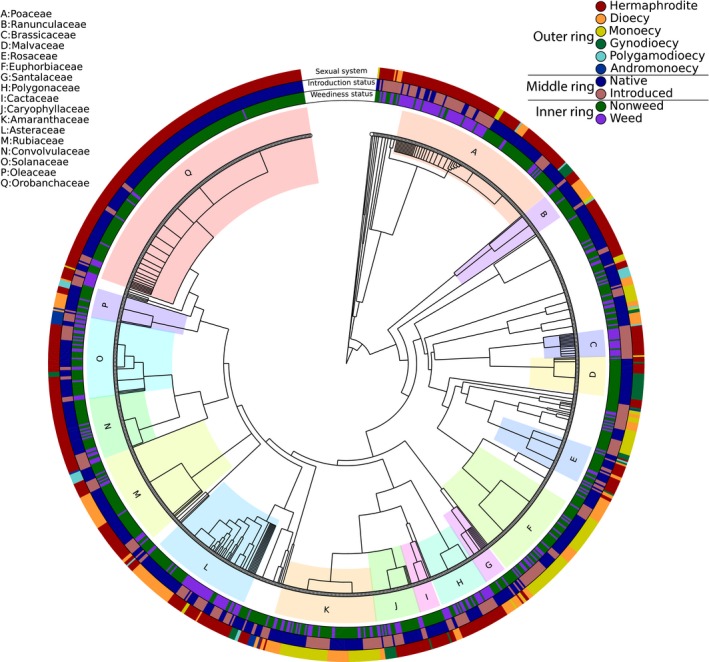
Phylogeny with sexual system (outer ring), introduction status (middle ring), and weediness status (inner ring) indicated for each species. The 17 most common families are shaded and labeled with letters

The merged dataset consisted of 1,077 species in 60 families, the most common being Orobanchaceae (18%), Poaceae (10%), Amaranthaceae (6%), Asteraceae (6%), Rubiaceae (6%), and Euphorbiaceae (6%). In the overall database, 61% of species were hermaphrodite, 17% dioecious, 13% monoecious, 4% gynodioecious, 2% polygamodioecious, and 1% andromonoecious (Fig 1; see Table 1 for sexual system descriptions). Many families were polymorphic for sexual system (47% of families), weediness (55% of families), or introduction status (52% of families; Figure 2; Figure S1).

**Figure 3:**
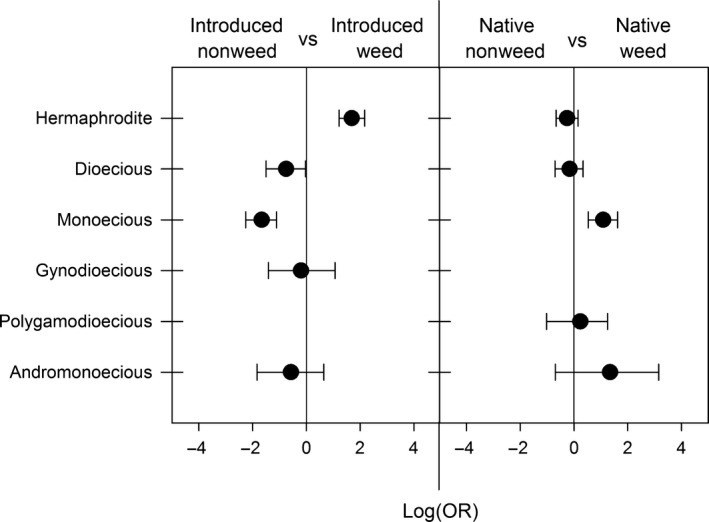
Results of binary logistic regressions comparing sexual systems between categories of species (e.g., introduced nonweeds vs introduced weeds). Each dot represents the log odds ratio (±95% confidence interval) of a particular model

In support of Baker's law, we found that introduced weeds are more likely to be hermaphroditic than introduced nonweeds (*p* < .0001; Figures 1 and 3); 78% of introduced weeds are hermaphroditic compared with 40% of introduced nonweeds. On the other hand, introduced weeds were less likely to be dioecious and monoecious than introduced nonweeds (*p* = .04, *p* < .0001; Figures 1 and 3). Over 60% of the introduced hermaphroditic weeds were found within four families: the Poaceae, Asteraceae, Brassicaceae, and Polygonaceae (Figure 2; Figure S1a).

Patterns in the native species were different than those in the introduced species: native weeds were more likely to be monoecious than native nonweeds (*p* < .0001; Figures 1 and 3), but the two groups of native species are equally likely to be hermaphroditic (*p* = .21; Figures 1 and 3). The monoecious native weeds tended to belong to the Amaranthaceae, Euphorbiaceae, Sapindaceae, and Poaceae (Figure 2; Figure S1b).

While we found that sexual systems were highly conserved within genera (Figure 2), the phylogenetic logistic regression provides support for the above taxonomic comparisons (Table 2), suggesting the patterns we uncovered are not due solely to phylogenetic relatedness. After removing the effect of phylogeny, the patterns found in the binomial regressions remained (Table 2). For introduced species, weeds were more likely to be hermaphroditic than were nonweeds (*p* < .0001) while the opposite was true for monoecy (*p* < .0001). On the other hand, for native species, weeds were more likely to be monoecious than were nonweeds (*p* = .002) and slightly less likely to be hermaphroditic (*p* = .04). Thus, our phylogenetic results support the hypothesis that sexual system varies with weediness status, whether native or introduced weed.

**Table 2:** Phylogenetic logistic regressions results for the effect of sexual system on weediness likelihood for native and introduced species. Alpha is the phylogenetic signal parameter with values >−4 indicating a phylogenetic signal

Sexual system	Introduced		Native	
	Log odds ratio	Alpha	Log odds ratio	Alpha
Hermaphrodite	1.56***	0.17	−0.50*	0.03
Dioecy	−0.58	0.04	−0.25	0.05
Monoecy	−1.56***	0.08	0.95*	0.04

*p < .05; ***p < .0001.

## 4 Discussion

Our phylogenetically controlled analysis of 1,077 species from 60 families showed that weedy plants, whether native or introduced to the USA, were more likely to exhibit sexual systems that promote uniparental reproduction compared with native and introduced nonweeds. Strikingly, native and introduced weeds exhibited different sexual systems: native weeds were enriched for monoecious species whereas introduced weeds were enriched for hermaphrodites. These results support Baker's law and the idea that certain sexual systems underlie the likelihood that a particular species will be identified as a weed.

### 4.1 Association between sexual system and weed status

The two different methodologies that we presented here—one based on taxonomy and the other correcting for phylogeny—showed that weeds differ from nonweeds in their distribution of sexual systems. In particular, we found an overabundance of hermaphrodites among introduced weeds. Although being a hermaphrodite does not guarantee that a species can self‐pollinate, the presence of male and female organs in each flower should increase the likelihood that a plant can reproduce in areas that may lack suitable mates. Surprisingly, we found a different pattern among native weeds, which were more likely than native nonweeds to be monoecious. We found no evidence that native weeds were more likely to be hermaphroditic compared with native nonweeds. Thus, our original expectation that weedy plants are more likely to have sexual systems that allow for uniparental reproduction appears to be correct, but native and introduced species differ in the sexual systems used to achieve this.

Previous studies examining the influence of sexual systems on weediness lead to conflicting conclusions. For example, Daehler (1998) compared different types of weeds (serious agricultural weeds vs widespread agricultural weeds vs. natural area invaders) on a global scale and found that family level rates of dioecy or monoecy did not differ among weed types. Sutherland (2004) found little evidence that US weeds were more likely to be hermaphroditic than nonweeds, but did find that invasive introduced weeds were more likely to be monoecious than were noninvasive introduced weeds. On the other hand, in an attempt to predict weediness, Reichard and Hamilton (1997) found that among woody plants in the USA having hermaphroditic flowers was associated with invasiveness. These conflicting results may be due to a variety of confounding differences, including the taxonomic groups used (all species vs only woody species), the regions covered (global vs. geographically restricted), and the particular comparison (weeds compared with nonweeds vs. introduced weeds compared with introduced nonweeds). Broader datasets that are a true sample of the region as well as using informative comparisons are needed to further clarify the role of sexual systems on weediness more broadly.

The difference that we uncovered in the predominant sexual system of native versus introduced weeds is likely due, at least in large part, to phylogeny. Notably, the majority of introduced weeds were from the Poaceae (many hermaphrodites), Asteraceae (many hermaphrodites and dioecious species), and Brassicaceae (mostly hermaphrodites), whereas the majority of native weeds were from the Amaranthaceae and Euphorbiaceae, many of which were either monoecious or dioecious. Our findings were similar to previous work from Kuester et al. (2014) showing introduced weeds in the USA to be significantly overrepresented by species belonging to the Poaceae, Asteraceae, and Brassicaceae, and that native weeds were overrepresented by species belonging to Amaranthaceae (among other families). Our broad interpretation of our data is that particular groups of taxa with sexual systems that promote uniparental reproduction are more likely to be successful colonizing weeds; however, under this interpretation, other traits shared among members of these families may underlie “weediness” (i.e., high growth rate and/or fecundity).

Perhaps the difference in predominant sexual system of the introduced and native weeds provides the best support for the idea that the sexual system is related to or promotes weediness—while we found that introduced and native weeds were enriched for different sexual systems, both of these sexual systems promote uniparental reproduction. For example, monoecious species are often considered to be functional hermaphrodites as they have both male and female flowers residing on a single individual (Richards, 1997). In this respect, a monoecious individual is more likely than a species with separate sexes to successfully produce progeny in the absence of other plants, especially if wind‐pollinated or if colonizing an area that does not lack pollinators. Thus, although the two different groups of weeds are overrepresented or enriched by particular families, those families that exhibit hermaphroditism—whether functional hermaphroditism through monoecy or hermaphroditism proper—are more capable of colonizing new areas and becoming classified as a weed.

### 4.2 What factors may explain the variation in sexual systems of native and introduced weeds compared with nonweeds?

The steps that influence the evolution of plant reproduction in colonizing species have recently been conceptualized into three main phases—dispersal, establishment, and potential subsequent evolution (Pannell, 2015; Richardson, Pyšek et al., 2000; Theoharides & Dukes, 2007). Because we compare groups of plants that are already established and weedy to plants that are established and nonweedy, our data are best interpreted in light of the postdispersal phases of establishment and potential subsequent evolution. During establishment, introduced species may be faced with both mate limitation and reductions in pollinator services if pollinators are rare or novel, and as such, the ability to autonomously self‐pollinate during the establishment phase would be highly beneficial. Members of the *Ficus* genus, for example, are primarily monoecious or gynodioecious and are pollinated by species‐specific wasp species (Nadel, Frank, & Knight, 1992). Species within this group have become invasive only when their specialist pollinators were accidently introduced (McKey & Kaufmann, 1991; Nadel et al., 1992; Ramirez & Montero, 1988). Thus, introduced species without a hermaphroditic sexual system might be less likely to become weedy because their specialized pollinators did not colonize with them. Native species, on the other hand, may be more likely to retain an association with their native pollinators during establishment, and thus may be less influenced by pollinator limitation. However, some studies suggest that most invasive species (included in our introduced weed category) are generalist‐pollinated (Richardson, Allsopp, D'Antonio, Milton, & Rejmanek, 2000) and are not more pollen‐limited than native species (but see Burns et al., 2011; Razanajatovo & Van Kleunen, 2016), in which case autonomous self‐pollination would not be beneficial.

Alternatively, and as above, the enrichment for functional hermaphrodites in weedy species could be due to selection on correlated traits during establishment that are ultimately responsible for the weediness status of a species. For example, wind pollination is associated with unisexual flowers (Friedman & Barrett, 2008) and dioecious species tend to be woody (Vamosi, Otto, & Barrett, 2003). Most studies (ours included) consider traits singly, but, as Baker clarified, there is no one “weedy” phenotype (Baker, 1965), and as such, considering only a single trait at a time limits our ability to identify these possible pathways. Strikingly, our previous work found that many traits are associated with both native and introduced weeds—annual life form, high growth rate, high fruit abundance, and high seedling vigor (Kuester et al., 2014). While selection for weediness could involve a host of traits, that we uncovered different predominant sex systems between introduced and native weeds, but sex systems that nonetheless may perform the same function (i.e., functional hermaphroditism), strongly suggests that traits which allow for uniparental reproduction are a key trait associated with weediness.

Our analysis, by necessity, assumes that hermaphroditic and monoecious species are able to reproduce uniparentally, that is, self‐pollinate. Although this may broadly be true, this simplification ignores a variety of mechanisms that limit selfing including self‐incompatibility, morphology (e.g., herkogamy), and developmental (e.g., dichogamy) mechanisms. To completely test the hypothesis that weedy species are more likely to be able to reproduce uniparentally, we would need extensive data on self‐compatibility as well as the circumstances under which selfing is possible (such as harsh environmental conditions or lack of outcross pollen). Given that large datasets of this kind do not yet exist, our results are a first pass at examining this hypothesis and suggest that uniparental reproduction is indeed an important factor in the development of weeds both native and introduced.

## 5 Conclusion

Here we provide evidence that sexual systems are an important characteristic related to plant weediness. Native and introduced weeds varied from nonweeds differently, with introduced weeds enriched for hermaphrodites and native weeds enriched for monoecious species. It is notable that introduced and native species appear to be quite taxonomically different, and yet, weeds of both groups are more likely to be functional hermaphrodites compared with their respective nonweeds. Overall, our results support the idea that weedy species are enriched for particular sex systems that allow for uniparental reproduction. These results show that Baker's law is reflected at the level of the sexual system, thus providing further evidence that uniparental reproduction is an important component of being either a native or introduced weed.

## Conflict of Interest

None declared.
