# Supplementary material for: Not all weeds are created equal: A database approach uncovers differences in the sexual system of native and introduced weeds
Source: Ecol Evol. 2017 Mar 18;7(8):2636–42. doi: 10.1002/ece3.2820 (PMC5395434; doi:10.1002/ece3.2820)

Sup Table 1. Multinomial logistic regression log odds ratios for sexual systems affect on weed status compared to Native non-weeds (hermaphroditism as reference level). Asterisks denote significance level.

|  | Introduced non-weed | Introduced weed | Native weed |
| --- | --- | --- | --- |
| Intercept | -1.977 | -0.916 | -1.839 |
| Dioecy | 0.062 | -1.304** | -0.025 |
| Monoecy | 1.977*** | -0.02 | 1.043** |
| Gynodioecy | 0.003 | -0.875* | na |
| Polygamodioecy | 0.185 | na | 0.335 |
| Andromonoecy | 2.67** | 1.427* | 1.434 |

Figure S1. Treemaps showing the relative proportion of sexual system for introduced (a) and native (b) species with brighter colors indicating weeds and darker colors non-weeds (NW).


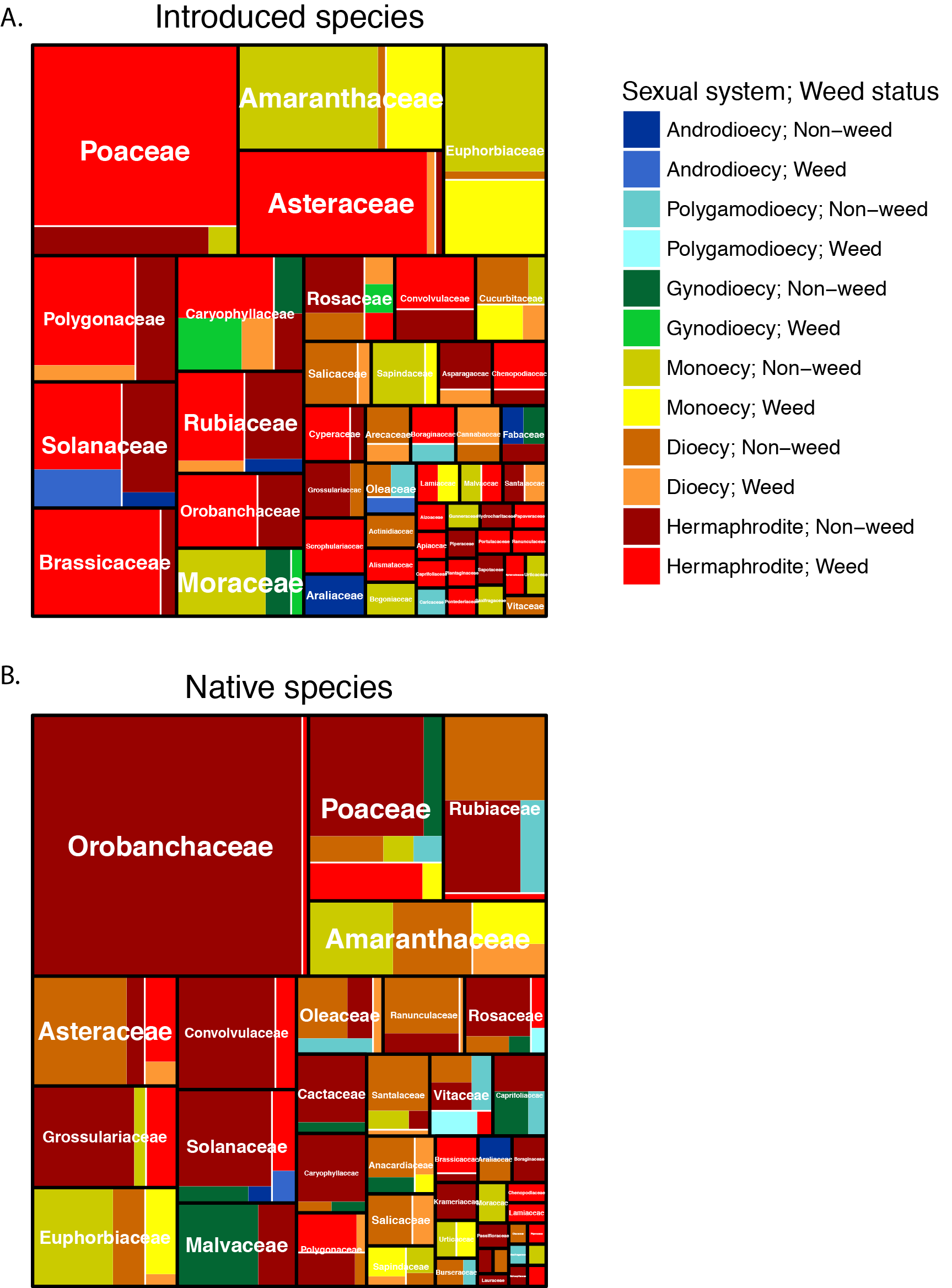

Supplement: Supplementary file 1 [file ECE3-7-2636-s001.docx]
